# Early warning indicator system for adolescent student mental health: theoretical construction of the relationship-belief-process-state model

**DOI:** 10.3389/fpsyg.2026.1887948

**Published:** 2026-07-29

**Authors:** Jianjun Zhong, Bingzhang Ci, Shanshan Zhang

**Affiliations:** 1 Research Center for Vocational Education Development, Key Research Base of Humanities and Social Sciences of Tianjin Higher Education Institutions, Tianjin, China; 2 School of Vocational Education, Tianjin University of Technology and Education, Tianjin, China; 3 China–Shanghai Cooperation Organization Vocational Education Cooperation Center, Tianjin, China

**Keywords:** adolescent mental health, early warning indicator system, high-risk configuration, relational characteristics-activity beliefs-congruent process-states (5R-5B-5P-5S) model, system configuration

## Abstract

**Background:**

Adolescent mental health problems are rising, but existing early warning systems are limited by shallow theory, poor cultural fit, and static risk-factor approaches.

**Aim:**

To construct a philosophically grounded, culturally adapted early warning indicator system for adolescent mental health in China.

**Methods:**

Based on two core propositions of Marxist humanism: “the essence of man is the ensemble of social relations” and “free, conscious activity is the species-character of man,” we developed the Relational Characteristics-Activity Beliefs-Congruent Process-Polar States (5R-5B-5P-5S) model. Each dimension was theoretically derived and operationalized.

**Results:**

The model specifies five relational characteristics (emotional connection, autonomy support, instrumental competence, fairness/reciprocity, shared meaning); five activity beliefs (success-failure, gain-loss, righteousness-benefit, self-other, effort-destiny); five congruent process indicators (awareness, acceptance, deconstruction, integration, implementation); and bipolar polar states (e.g., tension-relaxation, anxiety-peace, meaninglessness-fulfillment). A new early warning logic is proposed shifting from symptom checklists to systemic functional diagnosis, from state thresholds to high-risk configuration identification, and from generalized advice to targeted interventions.

**Conclusion:**

The 5R-5B-5P-5S model offers a theoretically robust, culturally conscious, and operationally feasible framework for adolescent mental health early warning, supporting a proactive health system in China.

## 1 Introduction

### 1.1 Policy requirements and practical challenges of adolescent mental health early warning in China

Currently, mental health problems among adolescents in China have become a major public health and social issue affecting family well-being, educational quality, and the nation’s future. Large-scale epidemiological surveys show that the prevalence of mental disorders among children and adolescents aged 6–16 in China has reached 17.5% (Li et al., 2022), and psychological distress is showing a serious trend of younger onset, concealment, and comorbidity (The Lancet, 2024). Against the backdrop of rapid economic and social transformation, intensifying educational competition, and deep penetration of digital media, traditional mental health work paradigms often rely on passive identification of overt symptoms and post-hoc intervention, making it difficult to achieve the strategic goals of early detection, early intervention, and early recovery. To this end, *Healthy China 2030 Planning Outline* and the *Special Action Plan for Comprehensively Strengthening and Improving Student Mental Health Work in the New Era (2023–2025)* explicitly call for shifting the focus from treating existing illnesses to preventing them, and building a new pattern of proactive early warning, tiered intervention, and systematic governance. The core challenge of this major transformation lies in how to construct an early warning indicator system that can scientifically reveal the essence of mental health, accurately identify early risks, and effectively guide multi-system coordination.

### 1.2 Theoretical paradigms of existing mental health early warning models and their limitations

Three major theoretical paradigms have gained widespread influence in the field of mental health early warning research: the biopsychosocial model, the ecological systems model, and the transdiagnostic framework. The traditional biopsychosocial model proposed by Engel (1977) broke through the limitation of the single biomedical disease-oriented perspective, arguing that illness originates from dynamic interactions among biological, psychological, and social systems—specifically, genetic predispositions and neurobiological foundations interact with cognitive patterns, emotion regulation, personality traits, and other psychological factors, as well as with family environment, social support, cultural background, and other social factors, to collectively shape an individual’s mental health status. This model constructed a holistic analytical framework encompassing biological vulnerability, individual psychological cognition, and social environmental factors for adolescent mental health development, and has become the dominant theoretical paradigm for contemporary mental health assessment and risk research (Bolton and Gillett, 2019). Although the model identifies genetic, biological susceptibility, cognitive biases, personality, social stress, and social support as effective predictors of disease and mental health, and has drawn on biological evolution and computational modeling to explain the causal mechanisms and dynamic changes of these factors in disease generation (Bolton and Gillett, 2019; Bolton, 2023; Abed et al., 2024; Känel, 2026), it nevertheless suffers, from the standpoint of causal mechanism research on disease and health, from insufficient systemic-philosophical logical self-consistency, inadequate attention to subjective experience in illness and disease, and insufficient emphasis on the causal role of human subjectivity in the interaction among biological, psychological, and social systems (Nelson et al., 2017; Benning, 2015; Tripathi et al., 2019). This has led to the BPSM being reduced to a static inventory of risk factors in early warning applications. Future efforts need to re-examine the causes, prediction, and treatment of disease and mental health from a person-centered perspective, focusing on causation as regulation and dysfunction as dysregulation (Bolton and Gillett, 2019).

Bronfenbrenner (1979) ecological systems model posits that the ecological environment in which individuals live is the primary source of influence on human development. The microsystems of an individual’s activities and interactions are nested within mesosystems, exosystems, macrosystems, and the chronosystem. Individuals engage in complex interactions with people, contexts, and time through proximal processes. Psychological crises do not arise from single causes; their emergence is closely related to the social-ecological environment and the interactions among relevant groups within that environment (Bronfenbrenner, 2004). Accordingly, this model proposes that ecological early warning of psychological crises should focus on the impact of individual personality and cognition, family functioning, school-family connections, community policies, and macro-cultural values on individual mental health. This early warning framework has been substantiated by research on the associations between psychological problems, mental disorders, and bio-ecological factors (Fadhlia et al., 2025; Thomsen et al., 2025; Dimova et al., 2026; Xu et al., 2026), establishing it as an important approach for attending to environmental risk factors. However, the bio-ecological early warning model focuses on ecological risk factors within multi-layered environmental structures but lacks in-depth investigation into the causal mechanisms through which proximal processes—the relationships between individuals and contexts, time, and significant others—lead to psychological crises and subsequently generate psychological problems and disorders. This remains a relatively static factor-based early warning approach, with insufficient exploration of the process mechanisms from cause to effect.

The transdiagnostic research framework addresses the tendency of traditional psychiatry to overemphasize symptomatic differences among different mental disorder categories, thereby amplifying between-category heterogeneity, while neglecting the fact that the correspondence between single-disorder classifications and biological mechanisms is extremely weak and that comorbidity implies shared risk factors and pathological mechanisms across comorbid types (Krueger and Markon, 2006). In response, the transdiagnostic paradigm advocates moving beyond rigid diagnostic labels and disease-specific research boundaries, focusing on identifying shared physiological, psychological, and social maladaptive mechanisms and core dysregulated processes that cut across the broad psychopathological spectrum, to inform pathological diagnosis based on shared generative mechanisms and common intervention targets based on these shared mechanisms (Dalgleish et al., 2020; Nature Mental Health, 2026). For example, it proposes classifying psychological disorders along spectrums according to shared maladaptive mechanisms, and identifying key pathogenic factors and core mechanisms of the psychopathological spectrum from the onset of psychological disturbance (Egan et al., 2011; Krueger and Eaton, 2015; Barlow et al., 2004; Norton and Roberge, 2017). This model simplifies complex pathogenic factors, mechanisms, and pathological types by tracing them to fundamental pathological mechanisms, aligning with the dimensional and continuously varying nature of psychiatric symptoms. It is not only suitable for early warning across the full spectrum of psychological distress—including subclinical, mild, comorbid, and chronically fluctuating populations—but also holds promise for achieving early warning at earlier stages of individual development through screening of potential pathogenic factors (Scott et al., 2024; Haas et al., 2025; Taquet et al., 2025). However, the major limitation of the transdiagnostic model is that its explanatory power for psychopathology is largely concentrated on phenotypic commonalities after the disorder has become established. It cannot systematically explain why individuals with the same vulnerability traits follow completely different developmental trajectories in different social-practical contexts (Nolen-Hoeksema and Watkins, 2011). This implies that for more accurate early warning, the transdiagnostic paradigm needs to attend to the dynamic expression of individual internal traits (psychological or neural) within the life-world, embedded social relations, and historical tensions in which individuals are situated (Achenbach, 2015).

Examining the three major models above reveals: the BPSM’s contribution lies in revealing the multi-dimensional influencing factors of mental health, yet it struggles to track the dynamic evolution of risk due to its failure to articulate causal interaction mechanisms among factors; the ecological systems model’s contribution lies in expanding the early warning horizon from the individual to multi-layered environments, yet it retreats to static factor listing due to the operationalization difficulties of “proximal processes”; the transdiagnostic model’s contribution lies in simplifying the complex pathological spectrum through shared mechanisms, yet it cannot explain why the same vulnerability traits lead to different outcomes across different social-practical contexts. These three limitations, while appearing to belong to different theoretical traditions, converge on a common root—all three are grounded in abstract, atomistic, and reductionist presuppositions about human nature, understanding psychological phenomena as products of individual internal traits, thereby severing the intrinsic connection between individual psychological activity and social practice and social relations, and weakening human subjectivity and the role of human agency in engaging with the world. In this sense, these three models are unable to achieve progressive, pre-clinical risk identification (Kirkbride, 2025; Öngür and Paulus, 2025).

### 1.3 Paradigm restructuring of early warning from the perspective of Marxist humanism and the positioning of this study

All the mainstream theoretical systems above share a homologous foundational defect: their conception of human nature is abstract and decontextualized, severing the intrinsic connection between individual psychological activity and social practice and social relations. In contrast, Marxist humanism offers a holistic, essential, and developmental interpretation of human beings, asserting that human nature is defined by social needs, free and conscious practical activity constitutes the human species-essence, and individual existence and development are fundamentally shaped by social relations through the individual’s adaptation and regulation of relationships with the external world on the basis of freedom and consciousness (Marx and Engels, 2012a,b,c). Accordingly, the root of adolescent psychological alienation lies in the sustained tension between individual developmental needs and social normative constraints, a tension that is continuously negotiated and reconstructed through daily social practice. In this process, free and conscious practical activity plays a crucial regulatory role in need satisfaction and environmental adaptation. Existing psychological models are unable to capture such practice-based, structural origins of psychological distress; they remain at the level of superficial, outcome-oriented risk assessment, and cannot achieve deep mechanistic explanation or precise early warning grounded in social relations, concrete agency, and world-oriented anticipation.

Beyond theoretical deficits, current early warning practices face dual constraints of cultural inadaptability and methodological limitations. At the cultural level, most widely used assessment tools have been developed within Western individualistic cultural contexts, emphasizing individual autonomy and linear competition, which conflict with the core values of relational harmony, intergenerational responsibility, and holistic social embeddedness advocated in Chinese culture (Cui and Wang, 2007). This cultural mismatch readily produces assessment bias, pathologizing interpersonally adaptive behaviors that are culturally appropriate while overlooking indigenous expressions of adolescent psychological distress. At the methodological level, mainstream early warning research relies excessively on cross-sectional data and static correlation analysis, conflating statistical associations with true causal mechanisms of psychological development. This paradigm cannot unpack the complete process through which multiple risk factors dynamically interact and accumulate over time to trigger psychological problems, inevitably yielding lagging and ambiguous warning outcomes that are difficult to translate into targeted preventive interventions (Glauser et al., 2020; Tate et al., 2020).

To address these challenges, this study grounds itself in the Marxist humanist theory of human nature, integrates the realities of Chinese culture and psychology, and innovates the theoretical paradigm of mental health generation mechanisms to systematically clarify the internal mechanisms and pathways through which adolescent mental health states are generated and transformed. On this basis, it aims to construct a set of early warning indicators capable of achieving proactive, precise, and ecologically valid early warning, thereby providing solid theoretical support and practical solutions for the scientific and systematic construction of adolescent mental health work in China in the new era.

## 2 Theoretical foundation and conceptual model: adolescent mental health from a Marxist humanist perspective

### 2.1 The essence of mental health: a philosophical reframing based on Marxist humanism

The construction of an early warning system for mental health must begin with a philosophical clarification of the essence of mental health and mental health states. The current mainstream psychological paradigm attributes mental health problems to individual internal neuronic dysfunction or mental trait defects, simplifying social relationships into external influencing factors. This leads early warning practice to overemphasize the improvement of individual cognitive coping and psychological resilience while neglecting the improvement of social psychological relationships, and overemphasize the recovery of individual internal dysfunctions while neglecting the development of people’s free and conscious psychological functions, ultimately failing to achieve a complete healthy state of human beings (Ryan and Deci, 2017; Wu et al., 2021).

Regarding the essence of human beings and its transformation, Marx developed a series of anthropological insights from the perspectives of human origins, the distinction between humans and animals, and the influence of social relations on human beings. Marxist humanism constitutes a complete logical chain of progressive layers: First, need is the fundamental nature of human beings. The German Ideology states, “Their needs, consequently their nature”—all human activities and psychological tendencies are rooted in multi-layered realistic needs (survival, communication, and developmental needs), and needs constitute the intrinsic motivation for all human behavior and psychological activities (Marx and Engels, 2012a,b,c). Second, free and conscious practical activity is the fundamental mode of human existence. Marx pointed out that the species-character of a species is determined by the character of its life-activity, and free conscious activity constitutes the species-character of man. The fundamental distinction between humans and animals lies in humans’ capacity to engage in objectifying practice that consciously transforms the subjective and objective world. Practice is the sole realistic path through which humans confirm themselves, construct social relations, and regulate inner cognition (Marx and Engels, 2012a,b,c). Third, the unity of dual scales is the unique species-essential characteristic of human practice. Animals are constrained solely by the single scale of their own species, whereas humans are capable of grasping the external law-like scale of all things (the other-scale) while simultaneously applying their own internal needs and potential scale (the self-scale) to the objects of practice. The coordinated unity of the dual scales is precisely the hallmark of human freedom (Marx and Engels, 2012a,b,c). Fourth, in reality, the essence of human beings is the ensemble of social relations. Abstract human beings detached from concrete interpersonal, institutional, and cultural relations do not exist. Human psychology, needs, cognition, and modes of practice are consistently shaped by the relational environment in which they are situated (Marx and Engels, 2012d). Fifth, human essence possesses historicity and developmental character in practice; alienation is the form in which human essence is distorted under specific social relations. In the Economic and Philosophic Manuscripts of 1844, Marx systematically expounded the theory of alienated labor, pointing out that under capitalist relations of production, human free and conscious labor—that is, the human species-essence—is alienated into forced, means-of-subsistence labor, resulting in human alienation from one’s species-essence, from other human beings, and from one’s own products of labor (Marx and Engels, 2012a,b,c). The essence of alienation lies in this: practical activities that should embody human free will and creativity are, under specific social relations, inverted into external forces that oppress and constrain human beings, threatening the organism and emotional well-being of human existence and development.

Marx pointed out that human beings are people in real social relations; animals construct only according to the scale and needs of their own species, whereas humans know how to produce according to the scale of any species and know how to apply their own inherent scale to objects; thus, humans also construct according to the laws of beauty (Marx, 2002). This means that true freedom is not laissez-faire under a single scale, but the integration of self-scale (internal needs and potential) and other-scale (social norms, others’ needs, objective laws) on the basis of consciousness. Therefore, the essence of mental health is a dynamic, congruent balanced state in which, through free and conscious practical activities under specific socio-historical conditions, individuals achieve the positive mutual construction and coordinated development of their psychological functions and social relationships. A mental health state is a self-organizing, healthy state in which psychological functions move toward the integration of the two scales in practice. Psychological problems such as anxiety, depression, compulsion, alienation, apathy, burnout, meaninglessness, nihility, and worthlessness are essentially psychological reactions to a rupture between the two scales—either falling into self-centered egotism or losing oneself as a subordinate to others—without achieving integration.

The generation of mental health states not occur in a vacuum. Marx’s assertion that humans are the ensemble of social relations reveals that psychological activities are always embedded in concrete historical social relations. Family, peers, teachers, social institutions, and cultural values constitute the relational ecological field for psychological generation. The quality of this field (e.g., supportiveness, autonomy, fairness, meaningfulness) directly regulates the space for individuals to achieve free consciousness, and social relations are relationships between real people, the most direct ecological field for mental health generation. Therefore, the generation mechanism of mental health states must be examined within an individual-relationship interaction framework (Tyler and Blader, 2003; Kirkbride, 2025).

The core process of mental health is the regulating practices between self and other scale through self-consciousness on the freedom. In the *Economic and Philosophical Manuscripts of 1844*, Marx profoundly elaborated on the dialectical movement of humans in survival and adaptation: “Man not only duplicates himself mentally in consciousness, but also actively and really duplicates himself in practice, thus intuiting himself in the world he has created” (Marx, 2000). This exposition reveals that the essence of survival adaptation is not passive compliance but a dialectical process of constantly negating existing states and creating new forms through objectifying activities. In practice, humans are both constrained by external conditions and actively change them through their own actions. This dialectical unity of passivity and activity constitutes the fundamental mechanism of survival adaptation. When individuals encounter relationship setbacks or practical difficulties (first negation), it is precisely the starting point of this dialectical movement: the inconsistency between external conditions and internal needs breaks the original psychological balance, forcing the individual to enter reflection and adjustment. This means that human freedom often must go through a sequence of predicament, awareness, acceptance, deconstruction, integration, and creation—a process that precisely reflects the dialectics of negation of negation, a transformation from conflict between scales to congruence between scales, and a necessary path to achieving a psychologically balanced and free state. The transformation begins with painful experiences triggered by relationship setbacks or practical difficulties (first negation), prompting individuals to become aware inwardly, breaking the fixation on original relationship patterns or self-cognitions; then through cognitive deconstruction and perspective-taking, identifying the crux of the problem; finally integrating experiences at a higher level, establishing new, more adaptive relationship patterns and self-understandings (second negation, i.e., negation of negation), achieving a leap in mental state. Whether individuals can maintain openness, awareness, and engagement between situational demands and personal values is precisely the cornerstone of mental health (Kashdan and Rottenberg, 2010).

Beliefs are stable and confident ideas and conscious actions formed by individuals in social practice on the basis of innate nature, concerning the self, others, the world, and their interrelations. In *The German Ideology*, Marx pointed out: “Consciousness can never be anything else than conscious being, and the being of humans is their real life process”; “Ideology is the imaginary expression of the living conditions of people in a certain way” (Marx and Engels, 2009). Beliefs are precisely the psychological sedimentation of this imaginary expression in individual cognitive structures, the subjective grasp and meaning construction by individuals of their own and social relations, practical conditions, and historical positions. From an evolutionary perspective, the belief system originates from integrating social feedback and interoceptive signals, constructing a cognitive niche for individuals to understand the world and predict the future, becoming a core mechanism for coordinating social interaction and guiding behavioral decisions (Dunn and Pooley, 2010). The formation and updating of beliefs depend on dynamic coupling changes in the prefrontal-limbic system of the brain, and this neural plasticity is precisely the biological basis for the flexibility of psychological processes (Kays et al., 2012; Seitz, 2022). The belief system plays a key mediating role in the process of free and conscious integration of the two scales.

The above analysis shows that mental health has a fundamental social relational embeddedness; its realization manifests as an active, free, conscious process; its transformation relies on a crucial cognitive mediational role of beliefs. These three points constitute the theoretical core of the entire model construction and also point the fundamental direction for early warning work: shifting from individual symptom monitoring to systemic assessment of relationships, beliefs, processes, and states.

### 2.2 The generative mechanism of adolescent mental health: a relationship-belief -process-state model

Adolescent mental health is deeply embedded in multi-layered ecological social relationships. Marx believed that need is a fundamental characteristic of human beings. Practical activities to satisfy needs according to basic needs and other scales always occur in real social relationships. A healthy mental state requires real social relationships to provide balanced psychological nutrition. Previous research in self-determination, humanistic, and transpersonal psychology has found that humans not only have needs for safety but also for competence and autonomy (Ryan and Deci, 2000), as well as needs for meaning and value (Frankl, 2006) and for fairness and reciprocity in interaction (Tyler and Blader, 2003). Therefore, real social relationships can provide security through emotional connection in interaction, developmental space through autonomy support, competence support through instrumental competence, justice experience through fairness and reciprocity, and value belonging through shared meaning. Systemic imbalance in relationship qualities will inevitably lead to spontaneous activation of negative emotions and negative self-defense systems, causing a systematic deviation in psychological development. In the Chinese context, the principal social contradiction is between the people’s ever-growing needs for a better life and unbalanced and inadequate development. The needs for a better life have been upgraded from the material level to the psychological level, manifesting as social psychological needs such as fairness perception and value identification (Xu, 2024), as well as positive psychological needs such as meaning and happiness (Peng, 2026), covering five core dimensions: achievement, fairness, morality, belonging, and autonomy. From the supply side, unbalanced and inadequate development, especially the insufficient supply of social relationships containing need-satisfying characteristics, will lead to imbalance in meeting these needs, resulting in relationship alienation, loss of psychological balance, and psychological distress such as achievement anxiety and distribution anxiety.

Beliefs are confirmed ideas and conscious action tendencies formed on the basis of innate nature through accumulation of life practice experiences, concerning the self, others, the environment, activities, and their interrelationships; they are a reflection of social being. Once formed, beliefs automatically guide and interpret individuals’ cognition, emotion, will, and behavior, occupying a mediating position between social relationships and psychological processes (Ellis, 1962; Rith-Najarian et al., 2019). In survival, individuals often face value choices between morality and self-interest, must set boundaries between what actions can change and what cannot, must define who they are in action, must face success and failure in actions, and must evaluate gains and losses from actions.

Faced with different cultures, individuals form different beliefs (Shweder et al., 2006). In the ecological survival mode of large river agriculture and the differential mode of social structure based on blood relations, Chinese culture has long attended to righteousness and benefit, effort and destiny, success and failure, gain and loss, group and self. Influenced by the traditional Chinese dialectical unity of yin and yang, Chinese culture has accumulated many insights into plain dialectical unity: the unity of righteousness and benefit (acting rightly necessarily brings benefit), harmony without uniformity, following one’s heart without transgressing rules, losing is a blessing, failure is the mother of success, and other confirmed ideas and action tendencies (Zhang, 2011, 2018). In educational and daily life realities, adolescent students, in pursuing satisfaction of their own needs, constantly deal with choices between righteousness and benefit in their actions, the boundary between the controllable effort and uncontrollable destiny, the experiential growth from success and failure, the evaluation of gains and losses from action investment, and the relationship between self and group in action. These beliefs act like a set of cultural filters and focusing lenses, continuously screening and interpreting experiences from the relational world, transforming them into specific value instructions that guide psychological processes, forming a cognitive-value coordinate system for Chinese adolescents to understand the world and guide action (Cui and Wang, 2007; Zhang, 2018). For example, a biased belief in success and failure can cause individuals to overfocus on threat signals; a negative belief in effort and destiny can weaken action motivation, and its neural basis highly overlaps with the circuitry of learned helplessness (Norris et al., 2025; Tafet and Ortiz Alonso, 2025).

Mental health does not lie in states per se, but in the psychological process of mental health. The smooth operation of the dynamic psychological process centered on the congruence between self-scale and other-scale is the essence of human mental health (Friston, 2010; Kays et al., 2012). Through the smooth operation of the congruent psychological process, individuals can regulate alienated self-scale or other-scale processes, achieve alignment of the two scales while maintaining smooth prediction, thereby returning from alienated states to the state of being a free and conscious person (Leidig, 2025; Shaw et al., 2026). These processes constitute the operational mechanism of free consciousness at the operational level: awareness is clear cognition of internal and external realities, the informational basis for free choice; acceptance is the tolerant bearing of experiential facts, creating psychological space for conscious coordination; deconstruction is the breaking and transcendence of rigid patterns, manifesting the subject’s creativity; integration is the synthesis and innovation of old and new experiences, forming a coherent self-understanding; implementation is the transformation of internal intentions into real action, completing the realization of practice. These five processes are interconnected and cyclical; their coordination, flexibility, and adaptability directly determine the functional level of mental health. Psychological distress is essentially a blockage, distortion, or rupture in some link of these processes (Kashdan and Rottenberg, 2010; Barrett, 2017a,b; Shaw et al., 2026).

## 3 Indicator system based on the relationship-belief-process-state model of mental health

### 3.1 Social relationships and their indicators

Marx believed that humans are the ensemble of social relations. The satisfaction of basic needs cannot be separated from a person’s real relationships. From the perspective of human beings, social relations are the characteristics that satisfy basic human needs; human alienation first begins with the alienation of basic needs in social relations. Therefore, abstract social relations can be decomposed into five inherent characteristics with clear psychological functions, which together constitute the social nutrition supply system for individual psychological development (Ryan and Deci, 2017; Hu et al., 2023). Social relationships differ because of their different psychological need-satisfying functions. The absence of these inherent psychological functions in social relations will lead to the dissatisfaction of basic human needs, which in turn will trigger either a one-scale alienation toward animals or an alienated psychological process of submission to other-scales.

Adolescence is a period of two overarching developmental imperatives: moving from dependence toward independence and from diffusion toward identity certainty. These imperatives generate five non-negotiable developmental questions: “Whom do I belong to?” (security base), “Who is in charge of my life?” (subjectivity), “What can I do?” (competence), “How does the world treat me?” (social trust), and “Why do I live?” (existential meaning). A relational characteristic is essential only if it directly answers one of these five questions; otherwise, it is ancillary. Applying this developmental-task criterion, the five characteristics presented below—emotional connection, autonomy support, instrumental competence, fairness and reciprocity, and shared meaning—emerge as the minimal set of relational nutrients indispensable for healthy adolescent development. Other commonly proposed characteristics, such as respect, intimacy, trust, validation, or communication, either serve as sub-dimensions of these five, function as enabling conditions rather than independent functions, or address developmental needs that are less fundamental than the five identified here. The following sections elaborate on each characteristic, tracing its theoretical foundations, psychological functions, and consequences of imbalance.

The five characteristics of social relations each have their own connotations, psychological functions, and mental health consequences when imbalanced. Emotional connection is the foundation of psychological safety. Its theoretical connotation refers to the authenticity, stability, and depth of emotional response, acceptance, and attachment in a relationship. It satisfies the individual’s most basic need for security and is the prerequisite for the development of other psychological functions. Through consistent emotional responses in social relationships, an internal working model of “the world is safe, I am loved” is established, serving as a secure base for exploring the outside world and coping with challenges. Imbalance in emotional connection: false (e.g., conditional love) or broken emotional connection leads to a lack of basic security, manifesting as anxious or avoidant attachment, affecting the establishment of all subsequent relationships (Tyler and Blader, 2003). Autonomy support is the nurturing space for subjectivity. Its theoretical connotation refers to the degree of respect and promotion of an individual’s independence, right to choose, and self-determination within a relationship. It is the external condition for the development of freedom as a human species-characteristic. By providing reasonable choice opportunities, respecting different opinions, and encouraging independent exploration, it cultivates intrinsic motivation, self-efficacy, and a sense of responsibility. Imbalance in autonomy support: excessive control suppresses subjectivity, leading to extrinsic motivation dominance, self-doubt, or rebellious opposition; excessive permissiveness may leave individuals without necessary guidance and boundaries (Deci and Ryan, 2000). Instrumental competence is the support system for practical ability. Its theoretical connotation refers to the effective help and resources a relationship provides in helping individuals master skills, solve problems, and achieve goals. It goes beyond emotional comfort to provide concrete methodological guidance and constructive feedback. Its mechanism of action is to build a sense of “I can cope with challenges” through timely help and effective feedback, enhancing practical problem-solving ability and frustration tolerance. Imbalance: long-term absence of instrumental support leads to learned helplessness and low self-efficacy; excessive spoon-feeding deprives individuals of opportunities to develop independent abilities. Fairness and reciprocity is the field of social justice experience. Its theoretical connotation refers to the perceived fairness of rights and obligations, giving and taking, and procedural fairness in a relationship. It concerns the individual’s basic trust in the social contract. Its mechanism of action is to establish the basic social belief that the world is just and cooperation is worthwhile through consistent rules and equal opportunities, promoting moral development and social adaptation. Imbalance: long-term exposure to injustice, exploitation, or discrimination breeds cynicism, distrust, or resignation and loss of dignity (Tyler and Blader, 2003). Shared meaning is the deep bond of spiritual belonging. Its theoretical connotation refers to the ability of two parties in a relationship to engage in deep dialogue, resonate, and co-construct meaning at the level of values, life goals, and existential meaning. It points directly to a person’s spiritual home. Its mechanism of action is to provide defense against existential anxiety and existential confirmation through spiritual dialogue and resonance, enhancing the sense of meaning in life, value identification, and spiritual belonging (Frankl, 2006; Waters et al., 2022). Lack of shared meaning leads to spiritual loneliness and value nihilism; even when surrounded by people, one still feels that no one understands one’s pain and pursuits. The five relationship characteristics form different configuration patterns in specific relationship domains (parent–child, teacher-student, peer), constituting the individual’s unique social psychological niche. For example, a family that overemphasizes instrumental competence (only caring about grades) while neglecting emotional connection (lacking emotional communication) may raise children who are high-achieving but emotionally distant.

The differential mode of Chinese society determines that adolescents’ social relationships also have a differential characteristic (Fei, 2015). For adolescent students, their social relationships take the family circle as the core, gradually develop teacher-student relationships, and then intimate friendships and peer relationships. Accordingly, the observation of basic need characteristics in each social relationship circle can be measured. In measurement, relationship type is the first-order indicator and relationship characteristic is the second-order indicator, as shown in Table 1.

**Table 1:** Relationship domains and relationship characteristics for social relationship measurement.

Relationship domain	Emotional connection	Autonomy support	Instrumental competence	Fairness and reciprocity	Shared meaning
Family circle (parents/caregivers)	Emotional acceptance and attention	Respect for choices	Guidance on learning difficulties	Consistency in family rules	Sharing and acceptance of dreams
	Unconditional love	Being listened to	Advice on interpersonal problems	Recognition of efforts	Exchange of life stories
	Physical touch security	Support for trying new things	Specific feedback on progress	Fair handling of conflicts	Shared meaningful activities
	Family belonging	Space for individualized development	Sharing of experiential wisdom	Keeping promises	Respect for values
Teacher-student circle (homeroom teacher/teachers)	Sense of safety when answering questions	Diversity of problem-solving methods	Clear explanation of difficult points advice on learning	Equal treatment	Stimulate interest in the subject
	Attention and inquiry about one’s state	Choice in group division	Advice on learning methods	Follow classroom rules	Explore the value of the content
	Genuine care for feelings	Freedom to question teaching content	Guidance on improving homework	Fairly recognize progress	Understand intergenerational pressure
	Tolerance of mistakes	Setting one’s own learning goals	Suggestions for coping with future uncertainty	Handle bullying justly	Inspire direction in life
Close friend circle (best friends/small group)	Safe disclosure of secrets	Respect for decisions	Advice on difficulties	Rough balance of giving and taking	Deep topic discussions
	Authentic self- expression	Joint discussion of activities	Sincere congratulations on achievements	Shared responsibility in conflicts	Shared sense of beauty in the world
	Response to companionship needs;	No coercion	Reminder and affirmation of strengths	Rotating emotional effort;	Support for inner pursuits;
	No rupture after conflict	Freedom in hobbies and interests	Sharing of information and resources	Balanced two-way listening	Experience of not being alone
Peer/classmate circle (class/club members)	NOT being excluded or isolated	Expression of ideas in groups	Sharing of learning materials	Fair distribution of opportunities	Sense of collective belonging and honor
	Being listened to when speaking	Choice of participation in activities	Mutual learning in teams	Reasoning in disputes	Motivation from shared goals
	Friendly group atmosphere	Tolerance of different interests	Recognition of one’s contributions	Reasonable task allocation	Sense of being an important
	Availability of help	Respect for one’s own pace	Value of classmates’ suggestions	Adherence to shared rules	Collective member value fit

### 3.2 Belief system and its indicators

The belief system is the cognitive conversion hub that transforms relational experience into psychological meaning; it is an individual’s probabilistic judgment of the connection between a subject and attributes or outcomes. According to ABC theory of emotion, when faced with the same event, what truly affects a person’s cognition, emotion, and action is often not the primary response to the event, but the second psychological fact based on the person’s beliefs about the event—the understanding, predictive judgments, and emotional reactions. Beliefs play a role in cognitive ecological positioning and navigation in human behavior and activities (Ellis, 1962; Albarracin and Pitliya, 2022; Golde et al., 2023). The belief system has a center-periphery hierarchical structure; core beliefs are the most stable, and once changed, they have a fundamental impact on the entire belief system.

Core beliefs derive their centrality from the fact that adaptation repeatedly confronts individuals with a set of fundamental problems that must be resolved. Adaptation unfolds within environments defined by uncertainty, risk, social norms, and the necessity of social cooperation—features that render every adaptive encounter fundamentally problematic. To adapt effectively, individuals must resolve five recurrent questions imposed by the structure of adaptive practice: whether one’s actions produce results (success-failure), whether resource investments yield acceptable returns (gain-loss), whether one’s conduct is justifiable to others (righteousness-benefit), where one stands relative to the group (self-group), and what lies within versus beyond one’s control (effort-destiny). For adolescents, these questions are not merely abstract but are encountered with heightened intensity and frequency. The transition from dependence to autonomy thrusts them into novel academic, social, and personal arenas where success and failure are acutely felt; the expansion of social networks and peer comparison intensifies gain-loss calculations; the emergence of moral reasoning and identity exploration makes righteousness-benefit judgments particularly salient; the dual strivings for belonging and individuation render self-group negotiations central; and the growing awareness of life’s contingencies demands a calibrated effort-destiny orientation. Beliefs formed about each dimension constitute the cognitive infrastructure that enables adolescents to process feedback, calibrate effort, maintain social standing, and sustain engagement across adaptive challenges (Ellis, 1962; Albarracin and Pitliya, 2022), ultimately supporting the two developmental mandates of autonomy attainment and identity consolidation (Havighurst, 1952; Erikson, 1968). We therefore focus on five belief domains of particular salience to adolescent adaptation—success-failure, gain-loss, righteousness-benefit, self-group, and effort-destiny—each elaborated below.

These five universal adaptive questions, however, are not resolved in a cultural vacuum. They are interpreted, weighted, and expressed through culturally specific meaning systems that shape what counts as success, what constitutes a justifiable gain, and where the boundaries of self and group are drawn. From the perspective of cultural psychology, the belief system carries a particular culture’s way of understanding the world. The Chinese understanding of basic propositions such as success-failure, gain-loss, righteousness-benefit, self-other, and effort-destiny forms a unique cognitive-value coordinate system (Yuwen, 2018). Core beliefs, in this framework, originate from the fundamental adaptive problems that individuals must resolve in the course of adaptation—problems that are not culturally arbitrary but structurally necessary. They differ from “irrational beliefs” in Western cognitive psychology; they are value-cognitive complexes with greater cultural depth and historical depth. For example, a Chinese adolescent’s belief about success and failure includes not only judgments of their own ability but also multiple cultural factors such as family expectations, social comparison, and face concerns. The beliefs about the state of congruence in free and conscious actions guided by the self-scale of basic needs and the other-scale can be summarized into the following five categories, each elaborated in turn.

Beliefs about success and failure refer to an individual’s fundamental understanding of success and failure, including how to define success and failure, how to view effort and results, and how to attribute the causes of success or failure. Success and failure are self-value positioning in a face-oriented society. Their cultural roots are deeply embedded in the Confucian view of achievement (“establishing virtue, establishing merit, establishing words”) and the Chinese cultural psychology of face. Success concerns not only the individual but also family honor and social evaluation. In the contemporary era of deep social media penetration, the comparison of success and failure faced by adolescents has extended from the real field to the virtual space, and this cultural pressure of “quantified self” further amplifies the impact of success-failure beliefs on mental health (Haidt, 2024). Success-failure beliefs involve core psychological processes such as self-efficacy, achievement motivation, and self-evaluation. They determine how individuals define success and failure, and how they view effort and results. Integrated success-failure beliefs view failure as feedback and a learning opportunity, emphasize process over outcome, and exhibit a growth mindset; biased success-failure beliefs equate failure with total denial of personal worth, pursue absolute perfection, and are extremely afraid of failure; negative success-failure beliefs view effort as useless, leave everything to fate, avoid challenges, and self-limit.

Gain and loss represent resource wisdom in a relational society, reflecting the logic of reciprocity in Chinese social interactions, traditional “give and take” wisdom, and the reality of resource competition in modern society. Gain-loss views involve cognitive processes such as decision anxiety, risk perception, future orientation, and opportunity cost calculation. People with integrated gain-loss beliefs understand the dialectical wisdom of “giving up gives gain,” can tolerate temporary losses for long-term value, and pursue win-win outcomes; those with biased gain-loss beliefs haggle over every ounce, fall into zero-sum thinking, worry about gains and losses, and feel anxious about immediate benefits; those with negative gain-loss beliefs avoid all choices and competition due to fear of loss, becoming passive and conservative.

Righteousness and benefit concern value coordination in a moral society. Inheriting the Confucian ethical tradition of the righteousness-benefit distinction, Chinese culture has developed layers of value dimensions such as “valuing righteousness over benefit; righteousness and benefit generate each other,” “righteousness before benefit,” and “using righteousness to restrain benefit.” The relationship between righteousness and benefit involves ethical psychological processes such as moral judgment, value conflict, conscience constraints, and inner harmony (Goyal et al., 2025; Skalski-Bednarz et al., 2025; Zheng et al., 2025). According to the trade-off between righteousness and benefit, three types are distinguished: those with integrated righteousness-benefit beliefs pursue the mutual reinforcement of righteousness and benefit, seeking balance between moral principles and personal interests in specific contexts; those with biased righteousness-benefit beliefs slide into extremes of either profit-mongering or moral absolutism, experiencing intense inner conflict; those with negative righteousness-benefit beliefs fall into moral relativism or nihilism, believing everything is empty and losing value pursuit (Tyler and Blader, 2003).

Self and group concern identity construction in a collective society, reflecting the dialectical tension between collectivist cultural tradition and the individualization trend in the modernization process. Psychologically, they involve social psychological processes such as self-identity, social identity, belonging needs, and individual expression. The traditional Chinese concept of “harmony without uniformity” allows individuals to achieve maximum satisfaction of both belonging needs (interpersonal harmony) and uniqueness needs (inner psychological harmony) when handling self-group relationships (Wang, 2010). Integrated beliefs adhere to “harmony without uniformity,” maintaining individuality while actively integrating into the group, with clear yet flexible boundaries; biased beliefs manifest as blind conformity with loss of self, or arrogant self-centeredness in opposition to all groups; negative beliefs lead to alienation and isolation, unable to integrate into the group nor establish an independent self.

Effort and destiny represent control cognition in the interplay of human effort and fate, integrating the traditional wisdom of “doing one’s best and accepting fate’s will” with the modern cultural expectation emphasizing individual control and achievement. They involve coping psychological processes such as sense of control, optimism-pessimism, psychological resilience, and attribution of setbacks. People with integrated effort-destiny beliefs exhibit a dialectical unity of active action and calm acceptance, with resilience and magnanimity; those with biased effort-destiny beliefs fall into the omnipotent fantasy of “man can conquer nature,” unable to accept failure and unpredictability; those with negative effort-destiny beliefs succumb to fatalism, believing everything is predetermined and effort futile. Modern neuroscience research on learned helplessness has shown that when individuals form the belief that effort is futile (negative effort-destiny belief), it is accompanied by reduced regulation of the raphe nucleus by the prefrontal cortex, thus weakening motivation and action (Maier and Seligman, 2016), providing biological evidence for the deep influence of beliefs on mental health.

These five categories have deep roots in Chinese cultural psychology. They differ from “irrational beliefs” in Western cognitive psychology; they are value-cognitive complexes with greater cultural depth and historical depth. For example, a Chinese adolescent’s belief about success and failure includes not only judgments of their own ability but also multiple cultural factors such as family expectations, social comparison, and face concerns. According to the main links involved in each belief category in activities, they can be further decomposed into second-order psychological process dimensions, as shown in Table 2.

**Table 2:** Core dimensions, observation content, and indicators of belief categories.

Category	Core dimension	integrated type manifestation	Biased type manifestation	Negative type manifestation
Success & failure	1. Goal orientation	Process-oriented/growth	Performance goal (outcome/evaluation oriented)	Avoidance goal, fear of failure
	2. Attribution style	Controllable factors (effort, strategy)	Uncontrollable factors (ability fixed, luck)	Externalized, generalized (“I never succeed”)
	3. Interpretation of failure	Information, learning opportunity	Denial of self-worth	Verification of expectation, reason to give up
	4. Definition of success	Multi-dimensional, personalized, includes growth	Singular, social comparison, outcome-oriented	Nihilistic (“success does not exist” or “pure luck”)
Gain & loss	1. Time frame	Long-term oriented, tolerates delayed gratification	Short-term oriented, eager for immediate reward	No orientation, avoids decision-making
	2. View of resources	Renewable, creatable, win-win	Scarce, fixed, zero-sum	Unrelated to self, unattainable
	3. Risk tolerance	Prudent risk-taking based on values	Either extreme risk aversion or blind risk-taking	No risk acceptable
	4. View of opportunity cost	Trades off for higher value, rational weighing	Anxious over all potential losses	Gives up all choices for fear of loss
Righteousness & benefit	1. Value ranking	Contextual balance with core principles	Absolute ranking (profit or righteousness absolute)	No stable ranking, follows the crowd
	2. Conflict resolution	Seeks creative third option	Intense inner conflict or extreme choice	Avoids conflict, numbly responds
	3. Moral emotion	Reparable guilt, peace of conscience	Intense shame or emotional numbness	Emotional apathy, no moral experience
	4. View of rules	Understands spirit, applies flexibly	Rigid adherence or arbitrary violation	Rules are useless, only power matters
Self & group	1. Self-boundary	Clear yet flexible	Blurred (engulfed) or rigid (isolated)	Unclear, difficult to establish
	2. Identity mode	Dual identification (individual and collective)	Overly self-loss or adversarial identity	Identity diffusion, no sense of belonging
	3. Relational expectation	Interdependent yet independent	Complete dependency or absolute independence	Distant, no expectations
	4. Conformity tendency	Rational choice based on value judgment	Blind conformity or reactionary opposition	Indifferent, non-participatory
Effort & destiny	1. Locus of control	Focuses on controllable, accepts uncontrollable	Exaggerated personal control (omnipotence) or full external control	Fully external, helpless
	2. Acceptance of uncertainty	Acknowledges uncertainty as part of life	Tries to control everything, cannot tolerate change	Overwhelmed by uncertainty, passive fatalism
	3. Belief in effort	“Doing one’s best” is valuable in itself	Effort guarantees success	Effort is useless
	4. Interpretation of setback	Temporary, local, transformable	Permanent, pervasive, catastrophic	Confirmation of fate’s injustice

### 3.3 Psychological process of free conscious realization and its measurement

The free conscious species-essence of human beings does not exist abstractly; it is realized in the continuous dialogue, calibration, and integration of the self-scale (internal needs, subjective experience) and the other-scale (external reality, social norms, others’ needs). The integration of the dual scales is essentially the resolution of contradictions between the self-scale (“I need, I believe, I feel”) and the other-scale (“the world demands, others need, objective laws obtain”). This resolution constitutes the fundamental process through which any organism makes adaptive decisions when confronted with multiple conflicting signals. From the basic logic of biological responding, any organism facing two conflicting information sources must sequentially complete the following functional processes.

First, both signals must be received simultaneously. If one attends only to the self-scale while neglecting the other-scale (or vice versa), no genuine conflict between the two scales emerges, and integration remains impossible. Awareness precisely satisfies this basic biological requirement—it brings both signals into the conscious field simultaneously, rendering them available for processing. This is a purely informational input function. Second, in the face of conflicting signals, the system must tolerate uncertainty without immediate collapse or premature action. In biological systems, conflicting signals inherently trigger defensive responses—fight, flight, or freeze. If the system cannot maintain functioning amid conflict, it regresses to control by a single scale. Acceptance sustains tolerance of the conflict signals—it enables the system to remain operational with both scales simultaneously present, rather than being driven by anxiety to shut down one of them. Third, in the face of conflicting signals, the system must break its original unitary response pattern. The organism initially possesses only one response repertoire (typically the fixed pattern dictated by the species-scale). Now confronted with two signals, the original pattern proves inadequate. Deconstruction consists in the inhibition and dismantling of the old pattern—it creates space for new response possibilities. Fourth, the system must find a new equilibrium between the two conflicting signals. This is not a simple choice between A and B, but the generation of a new pattern capable of responding to both signals simultaneously. Integration is precisely this creative synthesis—it generates new response solutions. Fifth, the new response solution must be tested in reality and adjusted according to feedback. The efficacy of any novel solution can only be validated through interaction with the real environment. Implementation is precisely this reality testing—it completes the adaptive cycle. These five functional processes constitute the minimal complete sequence of adaptive processing that any organism must execute when confronted with conflicting signals.

The realization of this process in humans is inseparable from prefrontal evolution. Evolutionary research on the prefrontal cortex has revealed that, analogous to the paralimbic prefrontal regions in rodents, the human paralimbic prefrontal cortex also subserves factual reactive inferences; analogous to the lateral prefrontal regions in primates, the human lateral prefrontal cortex also subserves factual proactive inferences; however, the human-unique frontopolar cortex possesses uniquely human capacities for counterfactual reasoning, hypothesis testing, metacognition, and monitoring and switching between multiple goals (Koechlin, 2014). Critically, the frontopolar cortex and its reorganization of connectivity with other brain regions enable humans to transcend the fixed framework within which animals can only respond to immediate stimuli according to their species-specific ecological niche (reactive inference) or predict the future based on past experience (proactive inference), and instead acquire the capacity to construct “what if” possibilities in imagination and to monitor and switch between multiple possible goals—thereby providing the neural foundation for free and conscious regulation based on dual-scale congruence. The connectivity between the prefrontal cortex and other brain functions provides the physiological basis for these functional processes (Menon, 2011): awareness requires the Salience Network’s (SN) interoceptive and exteroceptive monitoring; acceptance requires the Default Mode Network (DMN) and prefrontal-amygdala inhibition of emotional reactivity; deconstruction requires the Central Executive Network (CEN) and prefrontal-striatal inhibition of habitual responses; integration requires dynamic coupling among all three networks; and implementation requires the initiation of prefrontal-motor systems (Dosenbach et al., 2025). These five processes are the intrinsic logical requirements of dual-scale integration itself—to achieve the creative synthesis of “what I want” and “what the world needs,” one must sequentially complete the five irreducible psychological operations of awareness, acceptance, deconstruction, integration, and implementation. In terms of physiological foundation, prefrontal evolution—particularly the new functional organization formed by the frontopolar cortex after its evolution and its connections with other brain regions—provides the neural basis for free and conscious regulation processing between the two scales. Each process is elaborated in detail below.

Awareness provides the informational basis for congruence. Awareness clearly images internal states (self-scale: emotions, bodily sensations) and external stimuli (other-scale: others’ reactions, environmental demands). By seeing both scales through awareness, one avoids falling into subjective fantasy or external coercion, providing authentic and comprehensive data for congruence. Healthy awareness attends to both internal and external, imaging internal states while being aware of external information, characterized by openness, breadth, and present-moment focus, capable of simultaneously perceiving multi-dimensional information without being overwhelmed by any single piece. Awareness is the beginning of knowing, the starting point of emotional and motivational awareness. Awareness relies on the coordinated coupling of the awareness-attention network composed of sensory cortex (receiving information), insula (interoception), and anterior cingulate cortex (attention allocation), ensuring that internal and external information can be accessed with low distortion (Krönke et al., 2020; De Coninck et al., 2025).

Acceptance provides psychological space for congruence. Taking a non-judgmental, allowing attitude toward perceived internal and external realities (e.g., one’s own negative emotions, external setbacks) that do not meet expectations. Acceptance creates a buffer space for dealing with conflicts between self-scale and other-scale (e.g., “I do not like this but I have to do it”), reducing inner conflict caused by resisting reality (whether internal feelings or external facts), freeing psychological energy for creative problem-solving. Healthy acceptance is the ability to tolerate and contain unpleasant experiences, avoiding secondary pain such as anxiety about anxiety. Acceptance is the regulation of emotion and will, involving emotional tolerance and motivational adjustment (shifting from resistance to facing). Acceptance is related to the modulation of the ventromedial prefrontal cortex (emotion regulation) and certain functions of the default mode network (self-reference). Acceptance training (e.g., mindfulness) enhances the top-down regulatory coupling of the prefrontal cortex over the limbic system (especially the amygdala), reducing reactivity (Barrett, 2017a,b; Pastwa-Wojciechowska et al., 2021; Sezer et al., 2022).

Deconstruction is the source of flexibility in the congruence process. Deconstruction breaks automatic, rigid reaction patterns (e.g., “I must be perfect,” “others are always targeting me”) when facing self-other scale conflicts. When old thinking-behavior patterns cannot achieve congruence, deconstruction questions fixed self-scale cognitions and rigid interpretations of the other-scale, opening space for new possibilities and providing cognitive and behavioral flexibility. Healthy deconstruction is characterized by openness of thinking, ability to shift perspectives, and willingness to adjust habits. Deconstruction belongs to the category of higher-order knowledge (metacognition) and intention (intention adjustment); it is an active intervention in the automated chain of cognition, emotion, and will. Deconstruction processes highly depend on the executive control network of the dorsolateral prefrontal cortex, which can temporarily suppress the activity of dominant neural networks (breaking coupling), making it possible to reconfigure connections, reflecting the immediate function of neuroplasticity (Boschin, 2013; Kringelbach and Deco, 2024; Joyce et al., 2025).

Integration is the meaning creation of congruence. Through integration, new and old information and contradictory experiences (self-scale contradictions, self-other contradictions) are sorted and synthesized, generating new understandings, meanings, or solutions. The focus of integration is to creatively achieve congruence. Healthy integration is characterized by the ability to integrate experiences, the ability to create meaning, and the coherence of self-narrative; difficulty in integration leads to cognitive fragmentation, lack of meaning, and identity confusion. Therapeutic approaches such as narrative therapy and logotherapy aim to enhance integration ability. For example, integrating one’s own interest (self-scale) with social needs (other-scale) into a new career direction; integrating a failure experience into a growth narrative, producing a new value ranking, action strategy, or life story. Integration achieves the elevation of “knowing” through the formation of new schemas, new narratives, and new selves; transformation of emotion through the attribution of new emotional meaning; and new points of integration of intention through the determination of new goals. The integration process involves highly dynamic connectivity among the default mode network (self-reference, mental simulation, autobiographical memory), central executive network (logical reasoning), and salience network (value evaluation) (Tylén et al., 2015; Liang et al., 2016). Recent research shows that the ability to flexibly switch between brain states, i.e., “dynamic coupling,” is a key neural marker of mental health; integration difficulty corresponds to decreased switching efficiency among these networks (Doll et al., 2016; Arnsten et al., 2024; Jun et al., 2025).

Implementation is the transformation of congruence into reality. Implementation transforms the new intentions and plans formed through integration into concrete, effective actions that fit the context, adjusting flexibly, thereby turning the psychological congruence achieved internally into practical congruence in reality. Action itself is both the result of congruence and a way to test and revise congruence. It requires actions that are both faithful to the integrated self-scale (value direction) and flexibly adapted to new other-scales (real-world feedback) encountered during implementation. Implementation is the complete realization of intentions and plans but cannot be separated from the full support of metacognition (monitoring), meta-emotion (motivation), and meta-will (persistence) (Lyons and Zelazo, 2011). Implementation involves the coordinated work of the motor cortex, basal ganglia (habits and action initiation), prefrontal cortex (planning and monitoring), and the dopamine system involved in reward and motivation. A healthy action initiation-maintenance loop is key to the implementation process (Dosenbach et al., 2025) (see Table 3).

**Table 3:** Process dimensions and indicators.

Process dimension	Second-Order Indicators
Awareness (ability to clearly perceive internal and external information)	1.1 Body awareness (sensitivity and discrimination of interoceptive signals)
	1.2 Environmental awareness (breadth and depth of perception of external stimuli)
	1.3 Temporal awareness (perception and consciousness of time passing)
	1.4 Meta-awareness (awareness of the awareness process itself)
Acceptance (ability to non-judgmentally contain experiential reality)	2.1 Emotional acceptance (allowing and understanding negative emotions)
	2.2 Cognitive acceptance (tolerating contradictory thoughts and uncertainty)
	2.3 Relational acceptance (tolerating interpersonal differences and conflicts)
	2.4 Existential acceptance (calmly facing fundamental life propositions)
Deconstruction (psychological flexibility to break rigid patterns)	3.1 Cognitive elasticity (ability to think from multiple perspectives and avoid extremes)
	3.2 Belief updating (openness to revising beliefs based on new evidence)
	3.3 Problem reframing (cognitive ability to turn difficulties into opportunities)
	3.4 Perspective-taking (ability to step outside self-centeredness and observe objectively)
Integration (ability to create meaning and coherence)	4.1 Experience integration (weaving discrete experiences into a meaningful whole)
	4.2 Emotional wisdom (understanding deep meaning of emotions and using them for decision-making)
	4.3 Pattern recognition (ability to discover regularities and connections behind phenomena)
	4.4 Meaning construction (assigning personal and transcendent meaning to experiences)
Implementation (ability to transform intentions into effective action)	5.1 Action initiation (ability to overcome procrastination and start in time)
	5.2 Focus maintenance (ability to sustain attention and resist distractions)
	5.3 Flexible adjustment (flexibility to adapt to changes and adjust strategies)
	5.4 Motivation management (ability to maintain intrinsic drive and connection to values)

### 3.4 Mental health states and their measurement indicators

The essence of mental health states is a self-organizing state of free consciousness with congruence of the two scales. Mental health states, within the present theoretical framework, are not static categorical attributes but dynamic, self-organizing outcomes of the organism’s ongoing attempt to realize congruence between the self-scale (internal needs, subjective intentions, developmental strivings) and the other-scale (external objective demands, social norms, others‘needs) through self-consciousness for the freedom. Mental ill-health, by contrast, arises when this congruence fails—either the self-scale dominates at the expense of reality (egotistic alienation) or the other-scale dominates at the expense of subjectivity (submissive alienation)—triggering defensive dysregulation, allostatic overload, and a progressive constriction of adaptive flexibility (Seeman, 1959; Marx and Engels, 2012a,b,c). So, mental ill-health consists of mental activity states centered on the self or dominated by others. Pathological mental illnesses, neuroses, psychological disorders, or psychological distress are states of inner tension, anxiety, depression, and melancholy caused by defensive dysregulation when conflicts between self and external environment cannot be resolved through free and conscious congruence of the two scales. In contrast, mental health, in the state of congruence between self and other scales, manifests as creative, coordinated states of calmness, spontaneity, and relaxation, with satisfaction in real life (Vite et al., 2024; Sezer et al., 2025). However, in reality, the free and conscious practice of achieving congruence between self-scale and other-scale is not smoothly realized; there are always states where one dominates and the other is disadvantaged, as well as stalemate states. In this process, as the degree and duration of congruence failure increase, individuals experience frustration, boredom, shame, powerlessness, numbness. Simultaneously, to protect the self under the self-scale, they may even develop apathy, resentment, sarcasm, weariness, and indignation toward the other-scale. Consequently, mental health is best conceptualized as a continuum ranging from severe psychopathology and psychological distress, through ordinary psychological balance, to flourishing and self-actualization (Keyes, 2002; Gilmour, 2014). This continuum logic demands that measurement move beyond binary pathology-versus-health classifications toward a bipolar assessment that captures the dynamic quality of psychological functioning.

To provide a neurocomputational grounding for this bipolar continuum, Friston’s free-energy principle and active inference framework is integrated with Barrett‘s theory of constructed emotion. Under the free-energy principle, the brain is a hierarchical generative model that continuously generates top-down predictions about interoceptive, sensorimotor, and social outcomes, while minimizing bottom-up prediction errors (Friston, 2010; Seth and Friston, 2016; Barrett, 2017a,b; Heinz et al., 2019). Every self-other scale conflict is computationally instantiated as a prediction error which is a mismatch between prior expectations rooted in the self-scale and sensory evidence arising from the other-scale, which elevates variational free energy, heightens metabolic arousal (tension, anxiety), and mobilizes defensive allostatic responses (Clark, 2013; Seth and Friston, 2016). Conversely, when dual-scale congruence is achieved, it means that when subjective intentions are successfully integrated with environmental affordances and social expectations, then prediction errors are resolved, free energy is minimized, and the organism returns to a balanced energy budget, subjectively experienced as calmness, competence, interest, and hope. This dynamic precisely instantiates Marx’s dialectic of alienation and realization: congruence equals successful free-energy minimization (realization of species-essence), while rupture equals sustained high free energy and allostatic overload (alienation in practice).

Crucially, this prediction error minimization cannot be completed in a single computational step; it requires sequential processing across multiple levels of abstraction. Recent advances in predictive coding and neurocomputational psychiatry demonstrate that the brain‘s generative model is organized hierarchically, and adaptive functioning demands the progressive resolution of prediction errors from lower-order somatic and sensorimotor levels to higher-order conceptual, social, and existential levels (Shaw et al., 2026). Within the present dual-scale framework, five such levels are computationally irreducible: (1) somatic energy mobilization is the organism’s basic metabolic response to self-other prediction error; (2) instrumental efficacy confirmation is agentic evaluation of whether self-capacity matches external task demands; (3) value-laden meaning generation is higher-order conceptual integration of experience with intrinsic self-values; (4) interpersonal boundary negotiation is regulation of self-scale and other-scale within relational fields; and (5) existential self-world integration is global coherence judgment between individual being and universal reality. Each level provides the necessary prior constraints for the next: low-level allostatic errors (energy) must be addressed through action-oriented predictions (efficacy); if unresolved, they compel revisions of conceptual values (meaning), interpersonal strategies (relational), and ultimately the global self-model (existential) (Barrett, 2017a,b; Shaw et al., 2026). Persistent failure at any intermediate level propagates upward, eventually forcing global reorganization—computationally costly and manifesting as chronic high free energy, the neurocomputational signature of alienation. Conversely, sustained existential meaning can buffer lower-level errors via top-down modulation of the global prior, preventing distress escalation. Thus, the five-level sequence is not a theoretical imposition but a computationally necessary processing hierarchy grounded in the brain‘s predictive architecture.

Based on this theoretical logic, we operationalize mental health status for each of the five psychological function dimensions using a bipolar measurement strategy. For each dimension, we specify two contrasting emotional poles: one reflecting congruence success which is creative, coordinated, energetically balanced experiences and the other reflecting congruence failure which is defensive, dysregulated, energetically depleted experiences. This bipolar design captures the dynamic, non-static nature of mental health and provides an empirically tractable bridge between the philosophical concept of human essence realization and the neurocomputational language of predictive processing. Emotions, in this framework, are not subjective epiphenomena but direct interoceptive readouts of the organism‘s overall energy budget and hierarchical predictive success, is signals that indicate, at each level, whether the self-scale and other-scale are currently congruent or in rupture. Table 4 operationalizes this mapping across all five dimensions. For each level, the left column lists emotions characterizing successful congruence—indicating that prediction errors at that level have been minimized and energy budgets are balanced—while the right column lists emotions characterizing congruence failure—indicating unresolved prediction errors, elevated free energy, and allostatic overload.

**Table 4:** Emotional indicators of mental health states (bipolar).

Psychological function dimension	Congruence failure (defensive/dysregulated) emotions	Congruence success (creative/coordinated) emotions
Energy mobilization & regulation	Tension (energy over-concentration)	Calmness (balanced energy distribution)
	Unease (diffuse vigilance of energy)	Focus (directional concentration of energy)
	Anxiety (energy stuck in anticipated threat)	Anticipation (energy directed toward positive possibility)
	Irritation (energy mobilized but no outlet)	Flow (energy seamlessly connected to action)
Efficacy confirmation & feedback	Frustration (disconnection between effort and result)	Competence (match between ability and demand)
	Shame (social self-negation)	Pride (social self-affirmation)
	Embarrassment (minor social image damage)	Gratification (effort gains social recognition)
	Confusion (loss of direction in action)	Security (clear action path)
Meaning generation & connection	Uninterest (disconnection between activity and value)	Interest (resonance between activity and value)
	Boredom (repeated failure expectation)	Satisfaction (full experience of process)
	Emptiness (blurred inner value coordinates)	Fulfillment (time invested meaningfully)
	Disappointment (gentle frustration of unfulfilled expectation)	Connectedness (resonance with meaning system)
Relational mode & boundaries	Apathy (conscious withdrawal of emotional investment)	Curiosity (active expansion of cognitive boundaries)
	Numbness (closure of emotional feeling)	Vitality (natural flow of emotional energy)
	Alienation (psychological withdrawal from relational field)	Autonomy (maintaining self in relationship)
	Powerlessness (exhaustion of psychological energy)	Generosity (energy abundant and willing to share)
Existential confirmation & transcendence	Despair (closure of future possibilities)	Hope (openness of future possibilities)
	Worthlessness (fundamental denial of one’s right to exist)	Sense of worth (existence itself has value)
	Absurdity (awareness of meaninglessness in existence rules)	Awe (solemn feeling of existential order)
	Existential loneliness (incommensurable isolation of essence)	Oneness (fusion experience with blurred boundaries)

Table 4 thus serves as the outcome layer of of the 5R-5B-5P model. Its theoretical contribution lies in transforming abstract philosophical propositions (self-other scale congruence) and computational principles (hierarchical prediction error minimization) into concrete, bipolar emotional indicators that are empirically observable and clinically informative. In the subsequent section (Section 4), we articulate the causal pathway through which relational characteristics (5R) shape activity beliefs (5B), beliefs channel congruent processes (5P), and processes culminate in these bipolar emotional states (5S). This chain constitutes the model‘s core early-warning logic: rather than waiting for clinical symptoms to emerge, the system can identify high-risk configurations by detecting early disruptions in relationships and beliefs that, through hierarchical predictive propagation, will inevitably escalate into pathological emotional states if left unaddressed.

## 4 Relationships among the relationship-belief-process-state indicator system and early warning logic

### 4.1 Relationships among the relationship-belief-process-state early warning indicator system

When the quality of need-satisfying characteristics of social relationships declines, it triggers changes in beliefs, processes, and emotional states. First, from the perspective of basic psychological needs theory, relationship characteristics such as emotional connection, autonomy support, and instrumental competence correspond to the innate human needs for belonging, autonomy, and competence. Long-term deprivation of these needs triggers a “survival mode” alarm in the psychological system, forcing the system to reallocate resources to cope with threats—a process that naturally consumes resources for other psychological functions (Hobfoll, 1989). Second, according to cognitive dissonance theory, the core mission of the belief system is to provide a coherent explanatory framework for experiences. When external relational realities (e.g., betrayal) conflict with internal belief assumptions (e.g., the world is basically safe), the belief system must reconstruct to accommodate the inconsistent information, and this reconstruction tends toward defensive distortion (e.g., “no one can be trusted”) when resources are scarce (Ray, 2004). Third, the principle of limited cognitive resources dictates that psychological processes enter a functional compensation state when faced with relational stress. Continuous vigilance for relational threats leads to awareness coupling overload, consuming substantial attentional and executive control resources, reducing the efficiency of higher-order functions such as accepting new experiences, flexibly deconstructing, and deeply integrating due to insufficient resources (Muraven and Baumeister, 2000; Booth et al., 2017). These three mechanisms together make the chain reaction of declining relationship quality → distorted beliefs → blocked processes → worsening emotions a necessary outcome of system dynamics, not an accidental phenomenon.

Crucially, this chain reaction forms a self-fulfilling prophecy through predictive coding mechanisms. Modern neuroscience shows that the brain does not passively receive information but actively generates predictions and compares them with sensory input. When the belief system forms a distorted prediction such as “no one can be trusted,” it changes the weighting of information processing, paying more attention to clues that may confirm the prediction while ignoring or downplaying disconfirming evidence. In ambiguous social situations, this processing bias can lead neutral behaviors to be systematically misinterpreted as hostile (e.g., interpreting silence as contempt), and actions based on misinterpretation (e.g., defensive aggression or withdrawal) can evoke genuine negative reactions from others, thus confirming the initial distorted prediction. Social relationships profoundly affect individual emotional experience and physiological state through predictive coding mechanisms: when social exclusion occurs, the brain generates predictions about being excluded, thereby amplifying feelings of pain, forming a self-fulfilling cycle (Koban and Wager, 2016). Dynamic systems theory further points out that this self-reinforcing cycle is essentially an “attractor state” into which the psychological system falls; once there, the system spontaneously maintains this high-risk configuration, making it difficult to escape automatically (Finc et al., 2020; Scheffer et al., 2024; Poerio et al., 2025). This cycle reveals that distorted beliefs can create distorted perceptions of reality, and actions based on distorted perceptions can create distorted social realities (Villiger, 2023). This is the deep mechanism why, once relationship damage begins, it often accelerates and worsens, making it difficult to escape.

### 4.2 Social relationship-belief-process-state early warning logic

This model posits that psychological risk does not originate from a single indicator anomaly but from a high-risk configuration formed by specific relationship deficits, belief biases, and process blockages, which is essentially a systemic manifestation of hindered congruence realization between the two scales. The future core of mental health lies in shifting from static diagnosis to dynamic system monitoring, a judgment that provides top-level disciplinary consensus support for the early warning logic of this model (Öngür and Paulus, 2025; Feyaerts et al., 2026). The value of this logic is threefold:

First, achieving early risk identification. From theoretical deduction, the high-risk configuration is an integrated manifestation of systemic dysfunction in the relationship-belief-process chain. Relationship characteristic deficits are foundational issues in the psychological ecology, belief biases are the cognitive transformation of foundational issues, process blockages are the mechanistic manifestation of cognitive biases, and psychological symptoms are merely the external result of accumulated dysfunction across multiple links. It can therefore be inferred that by monitoring antecedent systemic indicators such as relationship characteristics and belief biases, early risk signals before symptom onset can be captured, creating a theoretically valuable window for preventive intervention. This breaks through the limitation of traditional symptom assessment, which can only capture overt problems, providing logical support for shifting the focus of intervention from crisis management to prevention.

Second, precisely locating the root of risk. By identifying high-risk configurations, it is possible to overcome the limitations of traditional symptom attribution and precisely locate the key nodes in the risk chain, providing a clear basis for targeted intervention. Clinical psychology research has shown that surface symptoms of psychological problems have the characteristic of heterogeneity—the same symptom may arise from different system configurations. This configurational analysis can effectively avoid the one-size-fits-all intervention mistake and improve intervention effectiveness.

Third, guiding targeted intervention. Designing intervention plans based on key abnormal nodes in the high-risk configuration can achieve precise interruption of the risk chain. According to the theoretical transmission chain of relationship → belief → process → state, first repair functional deficits in core relationships (e.g., improving family emotional connection quality through parent–child communication training), optimizing the psychological ecology at its root; second, conduct psychological process skill training in a safe environment (e.g., enhancing acceptance ability through mindfulness practice), repairing the realization pathway of two-scale congruence; finally, promote belief reconstruction through accumulated successful experiences (e.g., completing small goals to revise the belief that “effort is useless”), completing the cyclic optimization of system functions (Rith-Najarian et al., 2019). This intervention approach theoretically avoids the drawback of generalized, unfocused traditional intervention, providing a systematic framework for improving intervention effectiveness.

## 5 Future research

This study addresses the fundamental theoretical deficits of existing mental health early warning paradigms—namely, their abstract and decontextualized conception of human nature, their reliance on static factor enumeration, their inadaptability to the Chinese cultural context, and their inability to trace the causal pathways through which risk accumulates and transforms into psychological distress. By grounding the theoretical framework in Marxist humanism, the study systematically constructs a relationship-belief-process-state model, elucidating how relational characteristics shape beliefs, how beliefs guide congruent psychological processes, and how these processes converge into bipolar mental health states. The 5R-5B-5P-5S model provides, for the first time, a philosophically coherent, culturally grounded, and process-oriented framework that connects social-relational conditions through cognitive-belief mediation and psychological operations to emotional outcomes, thereby offering a theoretically transparent pathway for early warning that moves beyond symptom checklists toward systemic functional diagnosis. Beyond theoretical integration, this study contributes three novel insights. First, it establishes the primacy of relational need-supply characteristics as the foundational layer of mental health generation. Second, it introduces beliefs about success-failure, gain-loss, righteousness-benefit, self-other, and effort-destiny as cognitive mediators that transform relational experience into psychological processes, thereby accounting for the specific cultural logic of Chinese adolescent adaptation that mainstream Western models systematically overlook. Third, it proposes a bipolar, configurational early warning logic that shifts risk identification from isolated symptom thresholds to specific high-risk configurations of relationship deficits, belief biases, and process blockages, providing a theoretical foundation for targeted, mechanism-based interventions.

Despite its theoretical coherence and philosophical grounding, the present study has several limitations that should be acknowledged. First and foremost, this is a conceptual and theoretical paper, not an empirical investigation. The 5R-5B-5P-5S model, while systematically derived from Marxist humanism and supported by convergent evidence from developmental psychology, cognitive neuroscience, and cultural psychology, remains a theoretical proposition awaiting rigorous empirical testing. The proposed relationships among relational characteristics, beliefs, processes, and states—including the chain mediation hypothesis and the configurational risk hypothesis—have not yet been substantiated through empirical data. Second, the model’s generalizability across diverse adolescent populations requires further examination. While the five belief categories are culturally grounded in Chinese psychological realities, their applicability across different socioeconomic strata, geographic regions (urban vs. rural), and educational settings within China remains to be established. Third, the model’s emphasis on free conscious processes and dual-scale integration may not fully capture the role of unconscious or implicit processes that also influence mental health outcomes. Finally, the proposed early warning logic—shifting from symptom thresholds to high-risk configurations—requires empirical validation to determine whether configurational patterns indeed precede symptom onset and whether they can be reliably detected through the proposed assessment instruments. And thus requires empirical validation through the following approaches.

First, following established psychometric procedures, standardized instruments should be developed to measure each core dimension—relationship characteristics, belief categories, process indicators, and bipolar states—with item generation grounded in theoretical definitions, expert review for content validity, factor analysis for dimensional structure, and reliability testing, with measurement invariance assessed across demographic subgroups. Second, structural equation modeling should be employed to simultaneously test both the measurement model (confirming the hypothesized factor structures) and the structural model (testing the chain mediation hypothesis: relationship characteristics → beliefs → processes → states), examining direct and indirect effects within an integrated framework. Third, latent class or profile analysis should identify high-risk configurations characterized by specific combinations of relationship deficits, belief biases, and process blockages that form coherent, self-reinforcing risk patterns predicting poorer mental health outcomes. Finally, longitudinal tracking over multiple waves is essential to test the model’s dynamic assumptions—that high-risk configurations evolve over time, becoming entrenched through self-reinforcing feedback loops, or potentially disrupted by protective factors and timely interventions. Growth curve modeling and cross-lagged panel analyses can examine individual trajectories, reciprocal influences, and whether baseline indicators predict adverse developmental pathways. Fourth, longitudinal tracking over multiple waves is essential to test the model’s dynamic assumptions—that high-risk configurations evolve over time, becoming entrenched through self-reinforcing feedback loops, or potentially disrupted by protective factors and timely interventions. Growth curve modeling and cross-lagged panel analyses can examine individual trajectories, reciprocal influences, and whether baseline indicators predict adverse developmental pathways. Fifth, empirical refinement of cultural adaptability is required. While the current model considers cultural context at a theoretical level (e.g., differential mode, cultural rootedness of the five beliefs), empirical research is needed to reveal the specific mechanisms of cultural factors and validate the early warning efficacy of culture-specific beliefs, avoiding cultural universalism bias in theoretical construction. Cross-cultural comparative studies are recommended to test the factor structure and predictive validity of the five belief categories across different cultural backgrounds.

In addition, build an intelligent early warning platform integrating multi-source data: monitor relationship characteristics through social behavior indicators (e.g., interaction frequency, emotional valence), assess implementation processes through learning behavior indicators (e.g., focus duration, strategy adjustment), and assist in judging state indicators through smart band data (e.g., heart rate variability, sleep quality). Use algorithms such as random forests or graph neural networks to identify high-risk configurations, achieving intelligent management with real-time monitoring, automatic early warning, and precise intervention.
