# Correction: Early warning indicator system for adolescent student mental health: theoretical construction of the relationship-belief-process-state model

**DOI:** 10.3389/fpsyg.2026.1988119

**Published:** 2026-09-16

**Authors:** Jianjun Zhong, Bingzhang Ci, Shanshan Zhang

**Affiliations:** 1 Research Center for Vocational Education Development, Key Research Base of Humanities and Social Sciences of Tianjin Higher Education Institutions, Tianjin, China; 2 School of Vocational Education, Tianjin University of Technology and Education, Tianjin, China; 3 China–Shanghai Cooperation Organization Vocational Education Cooperation Center, Tianjin, China

**Keywords:** adolescent mental health, early warning indicator system, high-risk configuration, relational characteristics-activity beliefs-congruent process-states (5R-5B-5P-5S) model, system configuration

The email [zhongjianjun709@tute.edu.cn] was wrong.

The correct email is [zhongjianjun@tute.edu.cn].

The funder [Tianjin Education Commission Social Science Major Project (2022JWZD36)] was erroneously omitted.

The correct **Funding** statement reads:

The author(s) declared that financial support was received for this work and/or its publication. This work was supported by Tianjin Education Commission Social Science Major Project (2022JWZD36) and Tianjin University of Technology and Education Research Project (KYAQD202348).

The original version of this article has been updated.

